# Extracting Teeth

**Published:** 1865-02

**Authors:** 


### EXTRACTING TEETH.

Read before the Iowa State Dental Association, January 4th, 1865.

BY DR. COULSON.

GENTLEMEN OF THE PROFESSION:—I am called upon to prepare an Essay relative to extracting teeth.

Now, as there are no prescribed limits, you will pardon me if I should be eccentric, sometimes passing beyond the lines of demarkation, which are not well defined, but more frequently falling far short. The subject is a very interesting one, and one that deserves better efforts than I am able to give it.

The subject naturally divides itself into two divisions, first deciduous, and secondly, the adult or permanent. In considering the first, I would inquire, what are some of the pathological conditions requiring extraction. I will answer this by presenting a few cases. A child is presented with crowns broken or decayed even with the margin of the gums, high inflammation and swelling of the adjacent parts, restlessness, with fever and a disturbed condition of the nervous system. That is a severe case and demands immediate action. A case where the tooth of replacement is forcing the apex of the root anteriorly through the alveolus and gum and excoriating the lip. Where in the superior arch the absorption has not progressed and the tooth of replacement is being forced within the arch so that the edge will fall inside the lower arch. I believe that all teeth should be removed at a little before the tooth of replacement presents itself, from the fact that in the normal condition the deciduous crowns are removed before they are quite even with the margin of the gum; and I should not hesitate to extract in all such cases, and in all cases where there is violent constitutional disturbance; in fact, in all cases where in your judgment the present condition will be improved and the future unimpaired. Act collectedly, be careful in your diagnosis, study nature's laws, and you can hardly go wrong.

I am strenuously opposed to premature extracting. I have cases in my mind where the first teeth had been extracted prematurely, and either the germs of the second had been destroyed, or else the law governing second dentition had been so interfered with as to produce a cessation of the efforts of nature in that direction, and the consequence is no teeth, though the parties are far beyond the period of adolesence.

Gentlemen, I scarce like my subject; though I like to extract teeth, I do not like to do it all the time, I like to treat and fill them, and receive the pay; but as I write, it seems to me as though the words of my text were sounding in my ears—Extract teeth, extract teeth, but I hope it may not extract me from my subject.

There are many things to be taken into consideration in the extraction of teeth that must be left solely to the judgment and skill of the operator—the age, the temperament, the predisposition to inflammatory action, and many other conditions that should be carefully noted, and their action based on them. One word in reference to the mode of extracting and manner toward your patients. Try by all means to get the confidence of your little patient; be familiar, be pleasant, be sympathetic, for a child understands these things perfectly. Do not allow yourself to become the man of steel that will intimidate; make no unnecessary show of forceps, rather keep them concealed as much as possible; examine the tooth carefully; if loose, tell them so; if they ask if it will hurt, tell them it will; then contrast the momentary pain with hours or days; be honest with them; educate them to believe what you say is true to the letter, and you may have them so that they will submit cheerfully.

When they submit, having your forceps convenient and adapted, remove at once, then attract their minds from the operation as soon as possible, and the next time they will submit with but slight effort on your part.

If the tooth presents an unfavorable appearance, make your first effort doubly sure, for you will not get the second trial except by force, or if you do, it will be in rare cases.

I might here make a few remarks in regard to forceps. Get the best, become familiar with them, and then stick to them; for with even inferior tools you may with practice make finished work. If you become familiar with your forceps and take hold of another, though the difference is very slight, you will instantly detect it if you are a mechanic; if you are not, you may not.

In the extraction of the secondary teeth there is probably less persuasion necessary than in the first, though I have often found that patience coupled with persuasion has a beneficial effect.

If I were instructing a student, I should advise him to be honest with his patients; by all means tell them the truth, and if needs be the whole truth, and if their judgment is not sufficient, then after you have laid the case before them, then let them go and enjoy their dear rights to theii hearts' content, and when pain drives them to you the second time it may not require much of your eloquence to persuade them of your good intention.

It is a difficult matter to advise in the extraction of teeth in all cases, for here the judgment of the dentist must govern. In a majority of cases you can judge whether your patient will appreciate your efforts to save the tooth by judicious treatment and filling, and if you think not, after a proposition so to do, extract at once, for if you destroy the nerve and leave the tooth without a filling, it is folly, for in seventy-five cases out of a hundred, you will have the tooth to extract at some future time, and probably under very unfavorable conditions.

Instance, in periostal inflammation I would advise extracting in all cases where the following conditions exist: Where a tooth is necrosed; in the first stages of periostal inflammation; in ulceration from necrosis; in ulceration of long standing, with a disposition to involve adjacent parts; when a tooth has been broken loose by violence and is necrosed, and in many other cases where the judgment of the operator must govern, for it is not possible to lay down rules to govern all cases.

Preparatory to extracting a tooth, make a thorough examination of the walls with a sharp pointed instrument, probing the walls to satisfy yourself of their strength, for by so doing you may save yourself the unpleasant task of taking out a crownless root and your patients the pain of a second or third effort.

When placing the forceps on the tooth, remember the advice of Dr. White; if it is an upper tooth, push the forceps as though you were going to make them come out at the top of the head, drawing the inference that one successful effort is better than half a dozen unsuccessful ones, and finally reflect. Learn something every tooth you extract, cultivate your judgment from those cases that are passing daily under your notice.

*Iowa City, Ia., January 3d,* 1865.

### THE USE OF THE FILE.

Read before the Iowa State Dental Society, January 4th, 1865.

